# 4-Chloro-3-nitro­benzonitrile

**DOI:** 10.1107/S1600536808041330

**Published:** 2008-12-10

**Authors:** Bo-Nian Liu, Shi-Gui Tang, Hao-Yuan Li, Cheng Guo

**Affiliations:** a College of Science, Nanjing University of Technology, Xinmofan Road No. 5, Nanjing 210009, People’s Republic of China; b College of Life Sciences and Pharmaceutical Engineering, Nanjing University of Technology, Nanjing 210009, People’s Republic of China

## Abstract

In the title compound, C_7_H_3_ClN_2_O_2_, the Cl, C and N atoms are coplanar with the aromatic ring. In the crystal structure, weak inter­molecular C—H⋯O and C—H⋯N hydrogen bonds link the mol­ecules. The π–π contact between the benzene rings, [centroid–centroid distances = 3.912 (3) Å] may further stabilize the structure.

## Related literature

For a related structure, see: Sun & Wang (2006 ▶). For bond-length data, see: Allen *et al.* (1987 ▶).
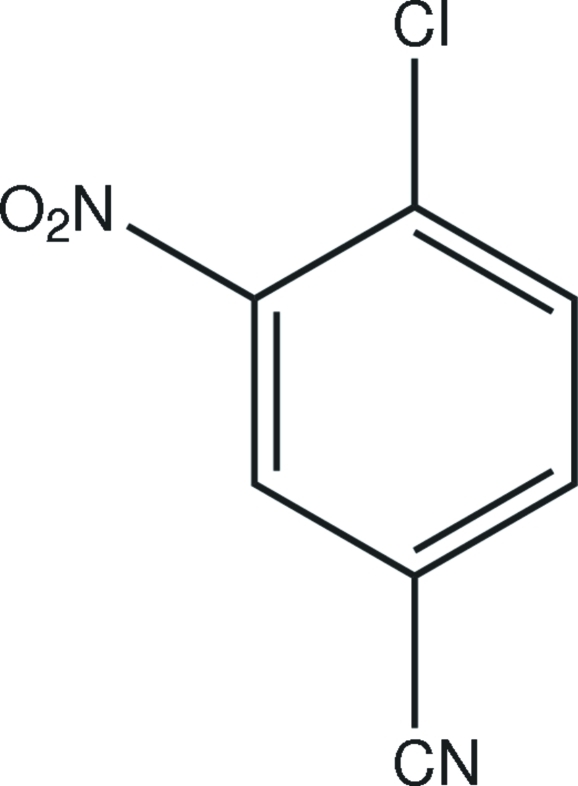


## Experimental

### 

#### Crystal data

C_7_H_3_ClN_2_O_2_
*M* *_r_* = 182.56
Triclinic, 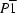

*a* = 7.2260 (14) Å
*b* = 7.7610 (16) Å
*c* = 7.7970 (16) Å
α = 110.27 (3)°
β = 91.86 (3)°
γ = 107.22 (3)°
*V* = 387.32 (18) Å^3^
*Z* = 2
Mo *K*α radiation
μ = 0.45 mm^−1^
*T* = 294 (2) K
0.30 × 0.20 × 0.10 mm

#### Data collection

Enraf–Nonius CAD-4 diffractometer
Absorption correction: ψ scan (North *et al.*, 1968 ▶) *T*_min_ = 0.878, *T*_max_ = 0.957
1540 measured reflections
1418 independent reflections
1000 reflections with *I* > 2σ(*I*)
*R*_int_ = 0.052
3 standard reflections frequency: 120 min intensity decay: none

#### Refinement

*R*[*F* ^2^ > 2σ(*F* ^2^)] = 0.073
*wR*(*F* ^2^) = 0.182
*S* = 1.00
1418 reflections
103 parameters
H-atom parameters constrained
Δρ_max_ = 0.27 e Å^−3^
Δρ_min_ = −0.33 e Å^−3^

### 

Data collection: *CAD-4 Software* (Enraf–Nonius, 1989 ▶); cell refinement: *CAD-4 Software*; data reduction: *XCAD4* (Harms & Wocadlo, 1995 ▶); program(s) used to solve structure: *SHELXS97* (Sheldrick, 2008 ▶); program(s) used to refine structure: *SHELXL97* (Sheldrick, 2008 ▶); molecular graphics: *ORTEP-3 for Windows* (Farrugia, 1997 ▶) and *PLATON* (Spek, 2003 ▶); software used to prepare material for publication: *SHELXL97* and *PLATON* ▶.

## Supplementary Material

Crystal structure: contains datablocks global, I. DOI: 10.1107/S1600536808041330/hk2597sup1.cif

Structure factors: contains datablocks I. DOI: 10.1107/S1600536808041330/hk2597Isup2.hkl

Additional supplementary materials:  crystallographic information; 3D view; checkCIF report

## Figures and Tables

**Table 1:** Hydrogen-bond geometry (Å, °)

*D*—H⋯*A*	*D*—H	H⋯*A*	*D*⋯*A*	*D*—H⋯*A*
C2—H2*A*⋯O1^i^	0.93	2.48	3.288 (7)	145
C5—H5*A*⋯N2^ii^	0.93	2.61	3.497 (7)	159

Symmetry codes: (i) 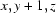; (ii) 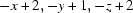.

**Fig. 1.**
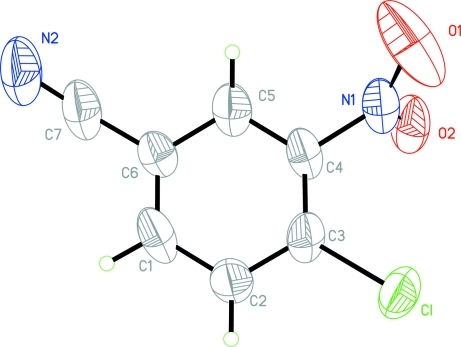
The molecular structure of the title molecule, with the atom-numbering scheme. Displacement ellipsoids are drawn at the 30% probability level.

**Fig. 2.**
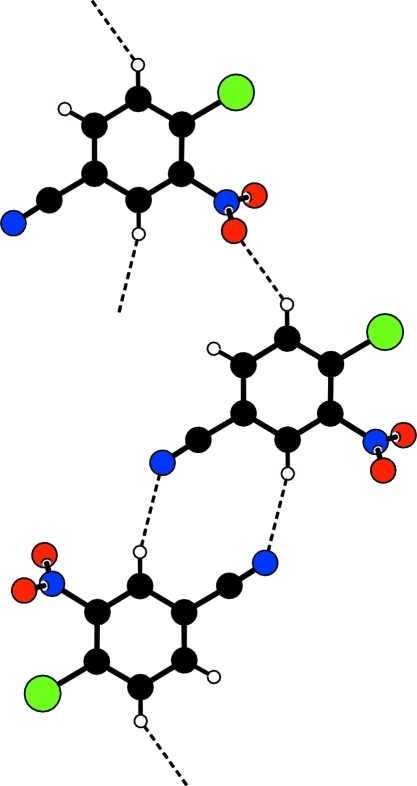
A partial packing diagram of the title compound. Hydrogen bonds are shown as dashed lines.

## supplementary crystallographic information

### Comment

Some derivatives of pyridine are important chemical materials. We report herein the crystal structure of the title compound.

In the molecule of the title compound (Fig 1), the bond lengths (Allen *et al.*,  1987) and angles are within normal ranges. Ring A (C1-C6) is, of course, planar. Atoms Cl, C7, N1 and N2 are -0.040 (3), -0.049 (3), 0.005 (3) and 0.036 (3) Å away from the plane of the benzene ring.

In the crystal structure, weak intermolecular C-H···O and C-H···N hydrogen bonds (Table 1) link the molecules (Fig. 2), in which they may be effective in the stabilization of the structure. The π-π contact between the benzene rings, Cg1—Cg1^i^ [symmetry code: (i) 1 - x, 1 - y, -z, where Cg1 is centroid of the ring A (C1-C6)] may further stabilize the structure, with centroid-centroid distance of 3.912 (3) Å.

### Experimental

For the preparation of the title compound, 4-chloro-3-nitrobenzamide (33.9 g, 0.17 mol) was suspended in phosphorus oxychloride (150 ml). The temperature was controlled at 333 K for 6 h, and then it was put into ice water (500 ml). It was filtered and the colorless precipitate was washed (yield; 28.2 g) (Sun *et al.*, 2006). Crystals suitable for X-ray analysis were obtained by slow evaporation of a methanol solution.

### Refinement

H atoms were positioned geometrically, with C-H = 0.93 Å for aromatic H and constrained to ride on their parent atoms, with U_iso_(H) = 1.2U_eq_(C).

### Crystal data

**Table:**

C_7_H_3_ClN_2_O_2_	*Z* = 2
*M**_r_* = 182.56	*F*(000) = 184
Triclinic, *P*1	*D*_x_ = 1.565 Mg m^−^^3^
Hall symbol: -P 1	Mo *K*α radiation, λ = 0.71073 Å
*a* = 7.2260 (14) Å	Cell parameters from 25 reflections
*b* = 7.7610 (16) Å	θ = 9–12°
*c* = 7.7970 (16) Å	µ = 0.45 mm^−^^1^
α = 110.27 (3)°	*T* = 294 K
β = 91.86 (3)°	Block, colorless
γ = 107.22 (3)°	0.30 × 0.20 × 0.10 mm
*V* = 387.32 (18) Å^3^	

### Data collection

**Table:**

Enraf–Nonius CAD-4 diffractometer	1000 reflections with *I* > 2σ(*I*)
Radiation source: fine-focus sealed tube	*R*_int_ = 0.052
graphite	θ_max_ = 25.3°, θ_min_ = 2.8°
ω/2θ scans	*h* = −8→8
Absorption correction: ψ scan (North *et al.*, 1968)	*k* = −9→8
*T*_min_ = 0.878, *T*_max_ = 0.957	*l* = 0→9
1540 measured reflections	3 standard reflections every 120 min
1418 independent reflections	intensity decay: none

### Refinement

**Table:**

Refinement on *F*^2^	Primary atom site location: structure-invariant direct methods
Least-squares matrix: full	Secondary atom site location: difference Fourier map
*R*[*F*^2^ > 2σ(*F*^2^)] = 0.073	Hydrogen site location: inferred from neighbouring sites
*wR*(*F*^2^) = 0.182	H-atom parameters constrained
*S* = 1.00	*w* = 1/[σ^2^(*F*_o_^2^) + (0.060*P*)^2^ + 0.880*P*] where *P* = (*F*_o_^2^ + 2*F*_c_^2^)/3
1418 reflections	(Δ/σ)_max_ < 0.001
103 parameters	Δρ_max_ = 0.27 e Å^−^^3^
0 restraints	Δρ_min_ = −0.33 e Å^−^^3^

### Special details

**Table:**

Geometry. All e.s.d.'s (except the e.s.d. in the dihedral angle between two l.s. planes) are estimated using the full covariance matrix. The cell e.s.d.'s are taken into account individually in the estimation of e.s.d.'s in distances, angles and torsion angles; correlations between e.s.d.'s in cell parameters are only used when they are defined by crystal symmetry. An approximate (isotropic) treatment of cell e.s.d.'s is used for estimating e.s.d.'s involving l.s. planes.
Refinement. Refinement of *F*^2^ against ALL reflections. The weighted *R*-factor *wR* and goodness of fit *S* are based on *F*^2^, conventional *R*-factors *R* are based on *F*, with *F* set to zero for negative *F*^2^. The threshold expression of *F*^2^ > σ(*F*^2^) is used only for calculating *R*-factors(gt) *etc*. and is not relevant to the choice of reflections for refinement. *R*-factors based on *F*^2^ are statistically about twice as large as those based on *F*, and *R*- factors based on ALL data will be even larger.

### Fractional atomic coordinates and isotropic or equivalent isotropic displacement parameters (Å^2^)

**Table:**

	*x*	*y*	*z*	*U*_iso_*/*U*_eq_	
Cl	0.08801 (17)	0.32257 (19)	0.5711 (2)	0.0767 (5)	
O1	0.3498 (8)	0.0413 (6)	0.7577 (7)	0.1159 (19)	
O2	0.2991 (5)	0.0203 (5)	0.4775 (6)	0.0819 (12)	
N1	0.3567 (5)	0.1130 (5)	0.6413 (6)	0.0590 (10)	
N2	1.0736 (7)	0.7724 (7)	1.0090 (8)	0.0942 (17)	
C1	0.6103 (7)	0.7170 (6)	0.8157 (7)	0.0652 (12)	
H1A	0.6681	0.8512	0.8556	0.077*	
C2	0.4139 (6)	0.6306 (6)	0.7290 (7)	0.0602 (12)	
H2A	0.3418	0.7057	0.7106	0.072*	
C3	0.3307 (6)	0.4305 (6)	0.6717 (6)	0.0560 (12)	
C4	0.4384 (6)	0.3252 (6)	0.7025 (6)	0.0502 (11)	
C5	0.6290 (6)	0.4094 (6)	0.7883 (6)	0.0581 (12)	
H5A	0.7001	0.3352	0.8108	0.070*	
C6	0.7137 (6)	0.6118 (6)	0.8413 (6)	0.0478 (10)	
C7	0.9133 (7)	0.7033 (7)	0.9310 (8)	0.0718 (15)	

### Atomic displacement parameters (Å^2^)

**Table:**

	*U*^11^	*U*^22^	*U*^33^	*U*^12^	*U*^13^	*U*^23^
Cl	0.0388 (6)	0.0651 (8)	0.1075 (11)	0.0117 (5)	0.0161 (6)	0.0143 (7)
O1	0.187 (5)	0.048 (2)	0.111 (4)	0.028 (3)	0.076 (3)	0.031 (2)
O2	0.064 (2)	0.047 (2)	0.098 (3)	0.0060 (16)	0.015 (2)	−0.007 (2)
N1	0.046 (2)	0.038 (2)	0.078 (3)	0.0103 (16)	0.027 (2)	0.005 (2)
N2	0.059 (3)	0.068 (3)	0.110 (4)	−0.004 (2)	−0.001 (3)	−0.001 (3)
C1	0.062 (2)	0.034 (3)	0.078 (3)	0.005 (2)	0.031 (2)	0.004 (2)
C2	0.046 (2)	0.048 (3)	0.079 (3)	0.018 (2)	0.022 (2)	0.011 (2)
C3	0.037 (2)	0.047 (2)	0.068 (3)	0.0102 (19)	0.027 (2)	0.003 (2)
C4	0.044 (2)	0.032 (2)	0.065 (3)	0.0098 (17)	0.033 (2)	0.0069 (19)
C5	0.043 (2)	0.042 (2)	0.071 (3)	0.0143 (19)	0.020 (2)	−0.001 (2)
C6	0.047 (2)	0.039 (2)	0.053 (2)	0.0128 (18)	0.0204 (19)	0.0112 (18)
C7	0.050 (3)	0.048 (3)	0.088 (4)	0.004 (2)	0.016 (3)	0.001 (3)

### Geometric parameters (Å, °)

**Table:**

Cl—C3	1.726 (4)	C4—C3	1.354 (6)
N1—O1	1.213 (6)	C4—C5	1.370 (6)
N1—O2	1.214 (5)	C5—C6	1.406 (6)
C1—H1A	0.9300	C5—H5A	0.9300
C2—C1	1.407 (7)	C6—C1	1.316 (6)
C2—C3	1.386 (6)	C6—C7	1.434 (7)
C2—H2A	0.9300	C7—N2	1.166 (6)
C4—N1	1.466 (5)		
			
O1—N1—O2	124.2 (4)	C2—C3—Cl	118.9 (4)
O1—N1—C4	117.9 (4)	C4—C3—Cl	121.5 (3)
O2—N1—C4	117.9 (4)	C4—C3—C2	119.5 (4)
C1—C2—H2A	120.9	C3—C4—N1	121.1 (4)
C1—C6—C5	120.8 (4)	C3—C4—C5	122.2 (4)
C1—C6—C7	120.3 (4)	C5—C4—N1	116.6 (4)
C2—C1—H1A	119.4	C4—C5—C6	117.8 (4)
C6—C1—C2	121.3 (4)	C4—C5—H5A	121.1
C6—C1—H1A	119.4	C6—C5—H5A	121.1
C3—C2—C1	118.3 (4)	C5—C6—C7	118.8 (4)
C3—C2—H2A	120.9	N2—C7—C6	176.8 (6)
			
C3—C2—C1—C6	0.4 (7)	C5—C4—C3—Cl	−177.5 (4)
C1—C2—C3—C4	0.9 (7)	N1—C4—C3—Cl	3.8 (6)
C1—C2—C3—Cl	178.2 (4)	C3—C4—C5—C6	−1.4 (7)
C3—C4—N1—O1	−120.7 (5)	N1—C4—C5—C6	177.3 (4)
C5—C4—N1—O1	60.6 (6)	C4—C5—C6—C1	2.6 (7)
C3—C4—N1—O2	58.9 (5)	C4—C5—C6—C7	−179.8 (4)
C5—C4—N1—O2	−119.8 (5)	C5—C6—C1—C2	−2.1 (7)
C5—C4—C3—C2	−0.3 (7)	C7—C6—C1—C2	−179.6 (5)
N1—C4—C3—C2	−179.0 (4)		

### Hydrogen-bond geometry (Å, °)

**Table:**

*D*—H···*A*	*D*—H	H···*A*	*D*···*A*	*D*—H···*A*
C2—H2A···O1^i^	0.93	2.48	3.288 (7)	145
C5—H5A···N2^ii^	0.93	2.61	3.497 (7)	159

Symmetry codes: (i) *x*, *y*+1, *z*; (ii) −*x*+2, −*y*+1, −*z*+2.
